# Psychological Flexibility as a Buffer against Caregiver Distress in Families with Psychosis

**DOI:** 10.3389/fpsyg.2017.01625

**Published:** 2017-10-04

**Authors:** Jens E. Jansen, Ulrik H. Haahr, Hanne-Grethe Lyse, Marlene B. Pedersen, Anne M. Trauelsen, Erik Simonsen

**Affiliations:** ^1^Mental Health Center Copenhagen, Frederiksberg, Denmark; ^2^Psychiatric Research Unit, Psychiatry Region Zealand, Slagelse, Denmark; ^3^Early Psychosis Intervention Center, Psychiatry East Region Zealand, Roskilde, Denmark; ^4^Region Hovedstadens Psykiatri, Copenhagen, Denmark; ^5^Faculty of Health and Medical Sciences, University of Copenhagen, Copenhagen, Denmark; ^6^Institute of Clinical Medicine, University of Copenhagen, Copenhagen, Denmark

**Keywords:** acceptance, first-episode psychosis, third-wave, family work, early intervention

## Abstract

**Background:** Research has shown that caregivers of persons with psychosis play an invaluable role in recovery, but unfortunately, often report high levels of distress. While cognitive models of caregiver distress have been well-supported, there is still limited knowledge of the psychological factors involved. Recent advances in cognitive behavioral therapy seem to converge on the importance of acceptance- and mindfulness based processes.

**Aim:** To examine the impact of psychological flexibility on caregiver distress in the early phases of psychosis, while controlling for known predictors of caregiver distress.

**Method:** Within a cross-sectional design, 101 caregivers of 38 persons with first-episode psychosis in a clinical epidemiological sample completed a series of self-report measures.

**Results:** A linear mixed model analysis found that, after controlling for caregiver socio-demographic factors, service user symptoms, drug use and global functioning, psychological flexibility was a significant predictor of caregiver distress.

**Conclusion:** Greater level of psychological flexibility in caregivers, seems to be related to lower levels of caregiver distress. This finding corresponds to studies within a broad range of emotional disorders. There may be important clinical implications in terms of facilitating the process of acceptance through interventions from the ‘third-wave’ or contextual cognitive behavioral therapies.

## Introduction

Research has shown that caregivers of persons with psychosis often play an invaluable role in recovery, and that a family environment characterized by warmth and low levels of criticism is associated with reduced risk of relapse (O’Brien et al., 2006; Lee et al., 2014). This is also reflected in service user perspectives on psychosis and recovery, that often highlight the importance of significant others (Jansen et al., 2015c). However, research also shows that caregivers report high levels of distress (Kuipers et al., 2005; Jansen et al., 2015b). Unfortunately, caregiver distress often feeds into ‘vicious circles’ (Kuipers et al., 2002): high levels of distress takes a toll on their mental resources, leading to higher levels of criticism and less energy to support the person with psychosis, which may exacerbate psychotic symptoms and increase the likelihood for relapse. This, in turn, increases caregivers’ feeling of hopelessness and distress. A number of studies have shown that structured family work reduces the risk of relapse, and improves the possibility of recovery in persons with psychosis (Pitschel-Walz et al., 2001; Pilling et al., 2002).

While cognitive models of caregiver distress have been well-supported, there is still limited knowledge of the psychological factors involved (Jansen et al., 2015a). Most of the studies focus on the service users, with comparably less focus on caregivers themselves. The lack of focus on caregiver distress, independent of the potential impact on the service user, has recently caused experts in the field to call for a separate caregiver support service (Kuipers, 2010). However, while we know a lot about effective strategies for supporting families facing psychosis, less is known about what makes some persons more distressed than others, or the ‘effective ingredients’ in these programs (Jansen et al., 2015a).

A number of treatment programs have been developed, mostly within a psychoeducational framework, based on cognitive behavioral principles, and their evidence in reducing relapse is considered robust (Pitschel-Walz et al., 2001; Pfammatter et al., 2006). Recent advances in cognitive behavior therapy seem to converge on the importance of acceptance- and mindfulness based processes when dealing with anxiety, depression and general distress. This is often referred to as the ‘third-wave’ of CBT (Hayes, 2004a) or contextual behavior therapy (Hayes et al., 2013) and examples include Dialectical Behavioral Therapy (DBT; Linehan, 1993), Metacognitive Therapy (MCT; Wells, 2000), Mindfulness-based Cognitive Therapy (MBCT; Segal et al., 2002) and Acceptance and Commitment Therapy (ACT; Hayes et al., 1999). The ‘third wave’ expands, rather that replace, traditional cognitive behavioral therapy, and share the common focus of helping the client relate to their thoughts differently, while de-emphasizing the need to change or ‘restructure’ specific cognitions (Tai and Turkington, 2009). In other words, the focus is more on the *process* rather than *content* of thinking. However, these processes have been less thoroughly examined within the field of family work. Jansen et al. (2014a) found that dysfunctional meta-cognitions, often related to increased worry and rumination, were related to caregiver distress, and that emotional over-involvement seemed to work as a mediator between these metacognitions and distress. In general, caregiver distress is beginning to emerge as a potential focus for mindfulness-based approaches (Epstein-Lubow et al., 2006; Minor et al., 2006). In a feasibility and acceptance study, Moorhead (2012) found that an 8 week mindfulness intervention was well-received by carers and service users, with promising effects on distress measures.

Acceptance and Commitment Therapy (Hayes et al., 2012b) targets experiential avoidance and a lack of flexibility and persistence in pursuing valued life directions. Experiential avoidance refers to an unwillingness to experience distressing emotions, or attempts to avoid internal experiences, including thoughts, feelings, memories, and physical sensations – even when doing so creates harm in the long-run (Hayes et al., 1999). The aim of ACT is to foster the opposite, namely ‘psychological flexibility,’ a process which is thought to be one of the central mechanisms of change in acceptance-based therapies. Psychological flexibility refers to an individual’s ability to connect with the present moment fully, to accept the thoughts, feelings and bodily sensations that ‘show up,’ and to persist in behavior that is in line with personal values (Hayes et al., 1999). Accordingly, distress is directly related to lack of psychological flexibility. Studies have found ACT to be effective in managing a number of disorders, including depression, anxiety, stress, psychosis, epilepsy, and pain (Hayes et al., 2006; Ost, 2008; Powers et al., 2009). Moreover, data from mediation analyses also suggest that psychological flexibility is the central therapeutic mechanisms behind the improvement (Ruiz, 2010; Vilardaga et al., 2013).

As a preliminary step toward examining the usefulness of acceptance-based approaches for caregivers of persons with psychosis, the aim of the current study was to examine the association between psychological flexibility and caregiver distress. We hypothesized that higher levels of acceptance would be associated with reduced caregiver distress, even when controlling for known predictors, including emotional involvement and subjective appraisal of burden, as well as service user characteristics such as symptoms and global functioning (Baronet, 1999; Tennakoon et al., 2000; Wolthaus et al., 2002; Addington et al., 2003; Boydell et al., 2013; Jansen et al., 2015b).

## Materials and Methods

### Design and Participants

The study had a cross-sectional design, and participants were relatives of persons with first-episode psychosis (age 18–35 years old). The sample is drawn from a larger intervention study and consists of follow-up data on this cohort. Service users were consecutively approached for inclusion upon receiving a schizophrenia spectrum disorder within Region Zealand in Denmark (population: 816,670) between April 2011 and April 2013. The inclusion criteria were: (1) meeting ICD- 10 criteria for schizophrenia spectrum disorders (F20–29, except F21), (2) first-ever psychiatric treatment for this diagnosis, (3) age 18–35. The only exclusion criterion was insufficient Danish skills for the interview to be completed. There were no additional inclusion/exclusion criteria for caregivers. The number of eligible individuals were 38 service users enrolled in the outpatient treatment service based on assertive community treatment principles (OPUS) together with 101 caregivers. Diagnoses were verified using operational criteria checklist (OPCRIT; McGuffin, 1991) by a clinical psychologist, MD or psychiatrist.

### Measures

#### Family Questionnaire (FQ; Wiedemann et al., 2002)

Expressed Emotion (EE) was assessed by the FQ, a 20-item self-report measure rated on a four-point Likert scale (‘never/very seldom,’ ‘seldom,’ ‘often,’ or ‘very often’). Participants are asked about everyday challenges with statements like ‘I have a tendency to neglect myself because of her/him’ and ‘she/he irritates me.’ The measure is divided into two subscales: emotional over-involvement (EOI) and critical comments (CC). EOI includes over-intrusive, self-sacrificing, over- protective behavior or exaggerated emotional response and over-identification with the service user; CC is defined as unfavorable comments on the behavior or personality of the service user (Vaughn and Leff, 1976). The items are scored from 1 to 4 yielding a maximum score of 40 in each subgroup. Caregivers are classified as high EE if they score 23 or greater on the CC subscale or 27 or greater on the EOI subscale. Wiedemann et al. (2002) found the FQ to have good psychometric properties including a clear factor structure, good internal consistency of subscales and good concurrent validity in relation to the widely used Camberwell Family Interview (Vaughn and Leff, 1976). They also found that the FQ displayed similar level of accuracy and higher sensitivity compared with the Five Minute Speech Sample (Magaña et al., 1986).

#### Experience of Caregiving Inventory (ECI; Szmukler et al., 1996)

Subjective appraisal of caregiving was assessed by the Experience of Caregiving Inventory (ECI), a 66-item self-report questionnaire. The questionnaire consists of 10 subscales: eight negative areas of caregiving (difficult behaviors, negative symptoms, stigma, problems with services, effects on the family, need to provide back-up, dependency and loss) and two positive (positive personal experiences and positive aspects of the relationship). Participants are asked how frequently they have thought about a series of issues during the last month. The items are scored on a five-point Likert scale with the response categories ‘never,’ ‘rarely,’ ‘sometimes,’ ‘often,’ and ‘nearly always.’ The maximum score for the combined negative subscales is 208 (higher scores equals more negative appraisals) and 56 for the positive subscales (higher scores equals more positive appraisals). The internal consistency and construct validity of the ECI have been found to be high (Szmukler et al., 1996; Joyce et al., 2000).

#### General Health Questionnaire 30 (GHQ; Goldberg and Williams, 1988)

The level of distress was measured using the General Health Questionnaire 30 (GHQ-30), which is a 30-item self-report questionnaire. For each item, the caregivers must rate symptoms on a four-point Likert scale ranging from 0 to 3, with a maximum score of 90 (‘better than usual/more so than usual,’ ‘same as usual,’ ‘less than usual,’ and ‘much less than usual’ for the positively worded items; and ‘not at all,’ ‘no more than usual,’ ‘more than usual,’ and ‘much more than usual’ for the negatively worded items). Higher scores signify greater distress. The scale has shown high internal consistency and good retest reliability, as well as concurrent validity with a number of other clinical assessments (Goldberg and Williams, 1988).

#### Acceptance and Action Questionnaire II (AAQ-II; Bond et al., 2011)

The Acceptance and Action Questionnaire II (AAQ-II) is a 10-item self-report measure, and is the most recent version of the widely used measure of psychological flexibility, experiential avoidance and acceptance of private experiences (Hayes, 2004b). Participants are asked to indicate agreement with statements (e.g., “It’s OK if I remember something unpleasant”) on a seven-point Likert scale (‘always true’ to ‘never true’). High scores reflect greater psychological flexibility. The AAQ has adequate internal consistency (Cronbach’s alpha = 0.84) and acceptable test–retest reliability (r’s = 0.81 and 0.79) (Bond et al., 2011).

#### Positive and Negative Syndrome Scale for Schizophrenia (PANSS; Kay et al., 1987)

Service user symptomatology were assessed with Positive and Negative Syndrome Scale for Schizophrenia (PANSS), a 30-item instrument, rated on a scale from 1 to 7. Participants are asked to report symptoms during the past week, and higher scores indicate more severe symptoms. PANSS is considered the most widely used instrument for symptoms in schizophrenia spectrum disorders in clinical trials. Psychometric properties have been found to be adequate (Peralta and Cuesta, 1994; Santor et al., 2007). PANSS were divided into positive, negative, disorganized, excitement, and emotional distress scores based on van der Gaag et al. (2006). Global functioning was measured using the DSM-IV’s Global Assessment of Functioning scale (GAF; American Psychiatric Association [APA], 1987).

### Procedure

Once the persons with psychosis were enrolled in the early psychosis intervention service (OPUS), they were approached for participation in the study. Following oral and written information about the study, and then written consent, caregivers were asked to fill out the four questionnaires and service users were asked to participate in a semi-structured diagnostic interview. All participants gave written consent to be contacted for 3-year follow-up interviews. All participants were debriefed following the completion of the questionnaires and the PANSS, but none of the respondents reported any adverse experiences in answering the questions. The Regional Committee for Research Ethics approved the study (reg.nr. 2008580020).

### Statistical Analyses

All statistical analyses were conducted using SPSS version 20 for Mac (IBM, Inc., Chicago, IL, United States). First, descriptive statistics of socio-demographic and clinical variables were calculated using means and standard deviations for quantitative variables and frequencies with percentages for categorical variables. Second, to accommodate for multiple caregivers per service user, and the possible correlation between them, a linear mixed model regression analysis was carried out to test the primary hypothesis.

## Results

### Sample Characteristics

The characteristics of the sample are presented in **Table 1**. From a total of 70 families, there were 101 caregivers between 28 and 72 years old (Median: 53) and 38 service users between 21 and 35 (Median: 23). A further 27 eligible service users did not participate, while 4 of these accepted for the caregivers to be involved. The majority of caregivers were female, living with the services users and employed.

**Table 1:** Characteristics of caregivers and service users.

Gender % female	% (n)	62.4 (63)
Age	M (SD)	52.16 (7.60)
Cohabitation	% (n)	60.4 (61)
Employment % yes	% (n)	71.3 (72)
Service user characteristics		
Gender % female	% (n)	33.3 (24)
Age	M (SD)	24 (3.23)
PANSS		
Positive symptoms	M (SD)	11.90 (4.96)
Negative symptoms	M (SD)	13.52 (6.38)
Disorganized symptoms	M (SD)	15.00 (4.50)
Excitement symptoms	M (SD)	11.16 (2.93)
Emotional distress	M (SD)	13.03 (4.90)
Global functioning, GAFs	M (SD)	57.37 (14.00)
Global functioning, GAFf	M (SD)	55.69 (13.61)


*N = 101 caregivers and 38 service users with first episode psychosis. M, mean; SD, standard deviation; PANSS, Positive and Negative Syndrome Scale for Schizophrenia; GAF, Global Assessment of Functioning Scale; GAFs, symptom score; GAFf, function score*.

### Questionnaires and Clinical Measures

The mean scores for GHQ was 28.35 (*SD*: 15.21). The mean scores for EOI was 23.14 (*SD*: 5.15) and for CC 18.23 (*SD:* 5.21), which is considered as low levels of expressed emotion. The mean score for ECI negative subscale was 110.79 (*SD*: 32.02) and ECI positive subscale was 39.75 (*SD*: 8.37). PANSS and GAF scores are presented in **Table 1**.

### Predictors of Caregiver Distress

The results of the linear mixed model analysis with distress (GHQ-30) as the main outcome are presented in **Table 2**. Higher scores on the AAQ-II, suggestive of greater psychological flexibility were significantly related to reduced caregiver distress (β = 0.79 [95% CI: 0.23 – 1.35]; *p* = 0.009). Results remained significant after controlling for caregivers’ gender, cohabitation and employment status, the level of symptoms, overall level of functioning and drug abuse in service users, and finally, other known predictors in caregivers such as subjective burden and the level of expressed emotion.

**Table 2:** Linear mixed model analysis with distress as dependent variable.

Predictors	β	95% CI	*p*
**Expressed emotion**			
FQ – Emotional Over-Involvement	1.01	0.41 to 2.44	0.150
FQ – Critical Comments	-0.04	-1.20 to 1.13	0.950
**Subjective appraisal**			
ECI – Negative subscale	-0.03	-0.24 to 0.18	0.754
ECI – Positive subscales	-0.58	-1.24 to 0.08	0.083
AAQ-II – Psychological flexibility	0.79	0.23 to 1.35	0.009
**Caregiver demographics**			
Gender	-5.24	-21.55 to 11.07	0.506
Cohabitation	2.64	-7.56 to 12.84	0.591
Employment	-14.75	-43.44 to 13.93	292
**PANSS (SU)**			
Positive component score	-1.55	-3.73 to 0.63	0.151
Negative component score	0.70	-2.28 to 0.89	0.365
Disorganized component score	0.46	-1.48 to 2.39	0.623
Excitement component score	3.29	-1.07 to 7.64	0.129
Emotional distress component score	0.48	-1.58 to 2.53	0.628
**Global functioning (SU)**			
GAFs	-0.68	-1.68 to 0.33	0.172
GAFf	1.03	-0.06 to 2.12	0.062
Drug abuse	0.71	-6.76 to 8.19	0.843


*N = 101. CIs, confidence intervals; FQ, Family Questionnaire; ECI, Experience of Caregiving Inventory; AAQ-II, Acceptance and Action Questionnaire II; PANSS, Positive and Negative Syndrome Scale for Schizophrenia; GAF, Global Assessment of Functioning Scale; GAFs, symptom score; GAFf, function score; SU, service-users*.

## Discussion

The current study examined the association between psychological flexibility and caregiver distress. Supporting our hypothesis, caregivers with higher levels of acceptance reported less distress. The result remained significant after controlling for caregiver demographic characteristics and known predictors such as emotional involvement and subjective appraisal of burden, as well as service user characteristics such as symptoms, global functioning, and drug abuse.

The positive association between psychological flexibility, as measured by the AAQ-II, and emotional distress, corresponds to findings from a range of studies, including depression, anxiety, stress, psychosis, epilepsy, and pain (Hayes et al., 2006; Ost, 2008; Powers et al., 2009). Psychological flexibility is considered the central mechanisms of change in these studies, as suggested by mediation analyses (Ruiz, 2010). This may be of importance since caregivers often report distress levels reaching thresholds for clinical significant symptoms such as depression, anxiety and post-traumatic stress disorder (Martens and Addington, 2001; Onwumere et al., 2011; Jansen et al., 2015b).

Studies suggest that caregivers in the early phase of psychosis, are extra vulnerable to distress, as they often experience grief and shock, and may have little prior knowledge of illness and the psychiatric system (Addington and Burnett, 2004). Finding ways to work through these difficulties demands time, energy and personal involvement from the whole family. Research has suggested that acceptance may be a central part of coming to terms with these changes. However, acceptance from the perspective of ‘third wave’ CBT, including ACT and mindfulness-based approaches, is not a passive response to suffering or incomplete recovery, but rather an active stance or ‘willingness’ to ‘be with’ the experience, ‘as it is,’ while engaging in or pursuing personally meaningful behavior (Hayes et al., 2012a; Jansen and Morris, 2016). For caregivers, thus, greater acceptance does not mean giving up hope for recovery. On the contrary, it means ‘making space’ for the anxiety, sadness, anger and grief, that is understandable and inevitable when supporting a person with psychosis. Mindfulness is a core component in acceptance-based therapies, and involves the cultivation of awareness, insight, wisdom, and compassion (Kabat-Zinn, 1990, 1994, 2003). In mindfulness, participants practice the skill of noticing distressing thoughts and feelings, holding such experiences in awareness, and meeting them with acceptance and self-compassion. This is a skill that can be learned and practiced, together with the other components of effective family work, including problem solving, psychoeducation and communication skills.

Some of the challenges families often deal with is loss of control, including a reduced sense of freedom and to some extent the relationship they used to have with their relative (Patterson et al., 2005). However, while the focus in research traditionally has been on caregiver ‘burden,’ the experience of caregiving is complex, and comprises both negative and positive experiences (Joyce et al., 2003; Jansen et al., 2014b). Caregivers also describe rewarding experiences, in addition to the pain, including increased closeness in the family and better understanding of other people’s suffering (Szmukler, 1996; Szmukler et al., 1996). This corresponds with the vast literature on post-traumatic growth, suggesting that people exposed even to the most traumatic events may perceive at least some good emerging from their struggle, including changes in self-perception, changes in interpersonal relationships, and a changed philosophy of life (Tedeschi and Calhoun, 1996; Park and Helgeson, 2006; Jordan et al., 2017). This also resonates with ideas within the recovery movement, which holds that you can live satisfying, meaningful lives, despite of experiencing distressing symptoms (Davidson et al., 2005). Taken together, expanding therapeutic interventions, to also encompass work on acceptance processes, as well as increasing hope, values and meaningfulness, may accommodate the challenges delineated above.

There are some limitations to this study. First, the cross-sectional design precludes firm causal inferences. It may be possible for instance, that lower levels of distress led participants to report being more accepting of difficult thoughts and emotions. Second, caregiver inclusion was based on consent by the service users, which represents a potential selection bias, as service users with better relationships with caregivers may have been more inclined to consent. Finally, our participants were White Danish adults; we do not know whether the findings generalize to other ethnic groups. There are also several strengths of the study. First, the comparably large sample drawn from a specialist early intervention service within a defined catchment area increases the likelihood of a representative sample. Second, the study adjusted for several confounders, which adds to the robustness of the predictor. Finally, two caregivers were included per service user in a multilevel analysis, which increases the number of males included compared to most studies.

With the limitations and strengths in mind, these findings may have important clinical implications. First, these initial findings are promising and add to the body of research showing that acceptance and mindfulness-based therapies can be used for people with psychosis and their caregivers (Chadwick et al., 2005, 2009; White et al., 2011; Jansen and Morris, 2016) as well as research on caregivers in other populations (e.g., Losada et al., 2015). Second, supporting caregivers in the process of accepting, or ‘making space’ for distress, while simultaneously addressing personally meaningful behaviors or values related to being a caregiver, may both instill hope and improve coping skills for difficult thoughts and feelings. This may broaden the intervention repertoire used in family support programs to better address the specific needs of the family.

## Author Contributions

JJ analyzed the data and wrote the first draft of the manuscript. All authors contributed to the writing of the manuscript, and agree with manuscript results and conclusions.

## Conflict of Interest Statement

The authors declare that the research was conducted in the absence of any commercial or financial relationships that could be construed as a potential conflict of interest.

## References

[B1] Addington J. Burnett P. (2004). “Working with families in the early stages of psychosis,” in *Psychological Interventions in Early Psychosis: A Treatment Handbook* eds Gleeson J. McGorry P. (Chichester: John Wiley and Sons) 99–116.

[B2] Addington J. Coldham E. L. Jones B. Ko T. Addington D. Addington A. (2003). The first episode of psychosis: the experience of relatives. *Acta Psychiatr. Scand.* 108 285–289. 10.1034/j.1600-0447.2003.00153.x 12956829

[B3] American Psychiatric Association [APA] (1987). *Diagnostic and Statistical Manual of Mental Disorders (DSM-III-R).* Washington, DC: American Psychiatric Association.

[B4] Baronet A. M. (1999). Factors associated with caregiver burden in mental illness: a critical review of the research literature. *Clin. Psychol. Rev.* 19 819–841. 10.1016/S0272-7358(98)00076-2 10520437

[B5] Bond F. W. Hayes S. C. Baer R. A. Carpenter K. M. Guenole N. Orcutt H. K. (2011). Preliminary psychometric properties of the Acceptance and Action Questionnaire-II: a revised measure of psychological inflexibility and experiential avoidance. *Behav. Ther.* 42 676–688. 10.1016/j.beth.2011.03.007 22035996

[B6] Boydell J. Onwumere J. Dutta R. Bhavsar V. Hill N. Morgan C. (2013). Caregiving in first-episode psychosis: social characteristics associated with perceived “burden” and associations with compulsory treatment. *Early Interv. Psychiatry* 8 122–129. 10.1111/eip.12041 23458284

[B7] Chadwick P. Hughes S. Russell D. Russell I. Dagnan D. (2009). Mindfulness groups for distressing voices and paranoia: a replication and randomized feasibility trial. *Behav. Cogn. Psychother.* 37 403–412. 10.1017/S1352465809990166 19545481

[B8] Chadwick P. Taylor K. N. Abba N. (2005). Mindfulness groups for people with psychosis. *Behav. Cogn. Psychother.* 33 351–359. 10.1017/S1352465805002158

[B9] Davidson L. O’Connell M. J. Tondora J. Lawless M. Evans A. C. (2005). Recovery in serious mental illness: a new wine or just a new bottle? *Prof. Psychol. Res. Pract.* 36 480–487. 10.1037/0735-7028.36.5.480

[B10] Epstein-Lubow G. P. Miller I. W. McBee L. (2006). Mindfulness training for caregivers. *Psychiatr. Serv.* 57 421–421. 10.1176/appi.ps.57.3.421 16525012

[B11] Goldberg D. Williams P. (1988). *A User’s Guide to the General Health Questionnaire.* Windsor, ON: NFER-Nelson.

[B12] Hayes S. C. (2004a). Acceptance and commitment therapy, relational frame theory, and the third wave of behavioral and cognitive therapies. *Behav. Ther.* 35 639–665. 10.1016/S0005-7894(04)80013-3 27993338

[B13] Hayes S. C. (2004b). Measuring experiential avoidance: a preliminary test of a working model. *Psychol. Rec.* 54 553–578. 10.1007/BF03395492

[B14] Hayes S. C. Levin M. E. Plumb-Vilardaga J. Villatte J. L. Pistorello J. (2013). Acceptance and commitment therapy and contextual behavioral science: examining the progress of a distinctive model of behavioral and cognitive therapy. *Behav. Ther.* 44 180–198. 10.1016/j.beth.2009.08.002 23611068 PMC3635495

[B15] Hayes S. C. Luoma J. B. Bond F. W. Masuda A. Lillis J. (2006). Acceptance and commitment therapy: model, processes and outcomes. *Behav. Res. Ther.* 44 1–25. 10.1016/j.brat.2005.06.006 16300724

[B16] Hayes S. C. Pistorello J. Levin M. E. (2012a). Acceptance and commitment therapy as a unified model of behavior change. *Couns. Psychol.* 40 976–1002. 10.1177/0011000012460836

[B17] Hayes S. C. Strosahl K. D. Wilson K. G. (1999). *Acceptance and Commitment Therapy: An Experiential Approach to Behavior Change.* New York, NY: Guilford Press.

[B18] Hayes S. C. Strosahl K. D. Wilson K. G. (2012b). *Acceptance and Commitment Therapy: The Process and Practice of Mindful Change.* New York City, NY: Guilford Press.

[B19] Jansen J. E. Gleeson J. Cotton S. (2015a). Towards a better understanding of caregiver distress in early psychosis: a systematic review of the psychological factors involved. *Clin. Psychol. Rev.* 35 56–66. 10.1016/j.cpr.2014.12.002 25531423

[B20] Jansen J. E. Haahr U. H. Harder S. Trauelsen A. M. Lyse H. Pedersen M. B. (2015b). Caregiver distress in first-episode psychosis: the role of subjective appraisal, over-involvement and symptomatology. *Soc. Psychiatry Psychiatr. Epidemiol.* 50 371–378. 10.1007/s00127-014-0935-8 25053150

[B21] Jansen J. E. Harder S. Haahr U. H. Lyse H.-G. Pedersen M. B. Trauelsen A. M. (2014a). The role of metacognitions in expressed emotion and distress: a study on caregivers of persons with first-episode psychosis. *Clin. Psychol. Psychother.* 22 525–532. 10.1002/cpp.1907 24889322

[B22] Jansen J. E. Lysaker P. H. Harder S. Haahr U. H. Lyse H.-G. G. Pedersen M. B. (2014b). Positive and negative caregiver experiences in first-episode psychosis: emotional overinvolvement, wellbeing and metacognition. *Psychol. Psychother.* 87 298–310. 10.1111/papt.12014 24038708

[B23] Jansen J. E. Morris E. M. J. (2016). Acceptance and commitment therapy for posttraumatic stress disorder in early psychosis: a case series. *Cogn. Behav. Pract.* 24 187–199. 10.1016/j.cbpra.2016.04.003

[B24] Jansen J. E. Wøldike P. M. Haahr U. H. Simonsen E. (2015c). Service user perspectives on the experience of illness and pathway to care in first-episode psychosis: a qualitative study within the TOP project. *Psychiatr. Q.* 86 83–94. 10.1007/s11126-014-9332-4 25464933

[B25] Jordan G. Pope M. Lambrou A. Malla A. Iyer S. N. (2017). Post-traumatic growth following a first episode of psychosis: a scoping review. *Early Interv. Psychiatry* 11 187–199. 10.1111/eip.12349 27189806

[B26] Joyce J. Leese M. Kuipers E. Szmukler G. Harris T. Staples E. (2003). Evaluating a model of caregiving for people with psychosis. *Soc. Psychiatry Psychiatr. Epidemiol.* 38 189–195. 10.1007/s00127-003-0618-3 12664229

[B27] Joyce J. Leese M. Szmukler G. (2000). The experience of caregiving inventory: further evidence. *Soc. Psychiatry Psychiatr. Epidemiol.* 35 185–189. 10.1007/s001270050202 10868084

[B28] Kabat-Zinn J. (1990). *Full Catastrophe Living.* New York, NY: Delta Trade Paperback.

[B29] Kabat-Zinn J. (1994). *Wherever You Go, There You Are: Mindfulness Meditation in Everyday Life.* New York, NY: Hyperion Books.

[B30] Kabat-Zinn J. (2003). Mindfulness-based interventions in context: past, present, and future. *Clin. Psychol. Sci. Pract.* 10 144–156. 10.1093/clipsy/bpg016

[B31] Kay S. R. Fiszbein A. Opler L. A. (1987). The Positive and Negative Syndrome Scale (PANSS) for schizophrenia. *Schizophr. Bull.* 13 261–276. 10.1093/schbul/13.2.261 3616518

[B32] Kuipers E. (2010). Time for a separate psychosis caregiver service? *J. Ment. Health* 19 401–404. 10.3109/09638237.2010.510155 20836686

[B33] Kuipers E. Bebbington P. Barrowclough B. (2005). “Research on burden and coping strategies in families of people with mental disorders: problems and perspectives,” in *Families and Mental Disorder: From Burden to Empowerment* eds Sartorius N. Leff J. Lopez J. Maj M. Okasha A. (Hoboken, NJ: Wiley) 217–234. 10.1002/0470024712.ch10

[B34] Kuipers E. Leff J. Lam D. (2002). *Family Work for Schizophrenia: A Practical Guide.* London: RCPsych Publications.

[B35] Lee G. Barrowclough C. Lobban F. (2014). Positive affect in the family environment protects against relapse in first-episode psychosis. *Soc. Psychiatry Psychiatr. Epidemiol.* 49 367–376. 10.1007/s00127-013-0768-x 24081324

[B36] Linehan M. M. (1993). *Cognitive Behavioral Treatment of Borderline Personality Disorder.* New York, NY: Guilford Press.

[B37] Losada A. Márquez-González M. Romero-Moreno R. Mausbach B. T. López J. Fernández-Fernández V. (2015). Cognitive–behavioral therapy (CBT) versus acceptance and commitment therapy (ACT) for dementia family caregivers with significant depressive symptoms: results of a randomized clinical trial. *J. Consult. Clin. Psychol.* 83 760–772. 10.1037/ccp0000028 26075381

[B38] Magaña A. B. Goldstein M. J. Karno M. Miklowitz D. J. Jenkins J. Falloon I. R. H. (1986). A brief method for assessing expressed emotion in relatives of psychiatric patients. *Psychiatry Res.* 17 203–212. 10.1016/0165-1781(86)90049-1 3704028

[B39] Martens L. Addington J. (2001). The psychological well-being of family members of individuals with schizophrenia. *Soc. Psychiatry Psychiatr. Epidemiol.* 36 128–133. 10.1007/s001270050301 11465784

[B40] McGuffin P. (1991). A polydiagnostic application of operational criteria in studies of psychotic illness. Development and reliability of the OPCRIT. *Arch. Gen. Psychiatry* 48 764–770. 10.1001/archpsyc.1991.01810320088015 1883262

[B41] Minor H. G. Carlson L. E. Makenzie M. J. Zernicke K. Jones L. (2006). Evaluation of a mindfulness-based stress reduction (MBSR) program for caregivers of children with chronic conditions. *Soc. Work Health Care* 43 91–109. 10.1300/J010v43n01 16723337

[B42] Moorhead S. (2012). Report of a feasibility study of a mindfulness group for clients, carers and staff of an early intervention in psychosis service. *Cogn. Behav. Ther.* 5 93–101. 10.1017/S1754470X13000019

[B43] O’Brien M. P. Gordon J. L. Bearden C. E. Lopez S. R. Kopelowicz A. Cannon T. D. (2006). Positive family environment predicts improvement in symptoms and social functioning among adolescents at imminent risk for onset of psychosis. *Schizophr. Res.* 81 269–275. 10.1016/j.schres.2005.10.005 16309893

[B44] Onwumere J. Bebbington P. Kuipers E. (2011). Family interventions in early psychosis: specificity and effectiveness. *Epidemiol. Psychiatr. Sci.* 20 113–119. 10.1017/S2045796011000187 21714356

[B45] Ost L.-G. (2008). Efficacy of the third wave of behavioral therapies: a systematic review and meta-analysis. *Behav. Res. Ther.* 46 296–321. 10.1016/j.brat.2007.12.005 18258216

[B46] Park C. L. Helgeson V. S. (2006). Introduction to the special section: growth following highly stressful life events–current status and future directions. *J. Consult. Clin. Psychol.* 74 791–796. 10.1037/0022-006X.74.5.791 17032084

[B47] Patterson P. Birchwood M. Cochrane R. (2005). Expressed emotion as an adaptation to loss: prospective study in first-episode psychosis. *Br. J. Psychiatry Suppl.* 48 s59–s64. 10.1192/bjp.187.48.s59 16055810

[B48] Peralta V. Cuesta M. J. (1994). Psychometric properties of the Positive and Negative Syndrome Scale (PANSS) in schizophrenia. *Psychiatry Res.* 53 31–40. 10.1016/0165-1781(94)90093-0 7991730

[B49] Pfammatter M. Junghan U. M. Brenner H. D. (2006). Efficacy of psychological therapy in schizophrenia: conclusions from meta-analyses. *Schizophr. Bull.* 32(Suppl. 1) S64–S80. 10.1093/schbul/sbl030 16905634 PMC2632545

[B50] Pilling S. Bebbington P. Kuipers E. Garety P. Geddes J. Orbach G. (2002). Psychological treatments in schizophrenia: I. Meta-analysis of family intervention and cognitive behaviour therapy. *Psychol. Med.* 32 763–782. 10.1017/S0033291702005895 12171372

[B51] Pitschel-Walz G. Leucht S. Bäuml J. Kissling W. Engel R. R. (2001). The effect of family interventions on relapse and rehospitalization in schizophrenia–a meta-analysis. *Schizophr. Bull.* 27 73–92. 10.1093/oxfordjournals.schbul.a006861 11215551

[B52] Powers M. B. Zum Vorde Sive Vording M. B. Emmelkamp P. M. (2009). Acceptance and commitment therapy: a meta-analytic review. *Psychother. Psychosom.* 78 73–80. 10.1159/000190790 19142046

[B53] Ruiz F. J. (2010). A review of acceptance and commitment therapy (ACT) empirical evidence: correlational, experimental psychopathology, component and outcome studies. *Int. J. Psychol. Psychol. Ther.* 10 125–162.

[B54] Santor D. A. Ascher-Svanum H. Lindenmayer J.-P. Obenchain R. L. (2007). Item response analysis of the Positive and Negative Syndrome Scale. *BMC Psychiatry* 7:66 10.1186/1471-244X-7-66 PMC2211479 18005449

[B55] Segal Z. V. Williams J. M. G. Teasdale J. D. (2002). *Mindfulness-Based Cognitive Therapy for Depression: A New Approach to Preventing Relapse.* New York, NY: Guilford Press.

[B56] Szmukler G. (1996). From family “burden” to caregiving. *Psychiatr. Bull.* 20 449–451. 10.1192/pb.20.8.449

[B57] Szmukler G. Burgess P. Herrman H. Benson A. Colusa S. Bloch S. (1996). Caring for relatives with serious mental illness: the development of the experience of caregiving inventory. *Soc. Psychiatry Psychiatr. Epidemiol.* 31 137–148. 10.1007/BF00785760 8766459

[B58] Tai S. Turkington D. (2009). The evolution of cognitive behavior therapy for schizophrenia: current practice and recent developments. *Schizophr. Bull.* 35 865–873. 10.1093/schbul/sbp080 19661198 PMC2728828

[B59] Tedeschi R. G. Calhoun L. G. (1996). The posttraumatic growth inventory: measuring the positive legacy of trauma. *J. Trauma Stress* 9 455–471. 10.1007/BF02103658 8827649

[B60] Tennakoon L. Fannon D. Doku V. O’Ceallaigh S. Soni W. Santamaria M. (2000). Experience of caregiving: relatives of people experiencing a first episode of psychosis. *Br. J. Psychiatry* 177 529–533. 10.1192/bjp.177.6.529 11102328

[B61] van der Gaag M. Hoffman T. Remijsen M. Hijman R. de Haan L. van Meijel B. (2006). The five-factor model of the Positive and Negative Syndrome Scale II: a ten-fold cross-validation of a revised model. *Schizophr. Res.* 85 280–287. 10.1016/j.schres.2006.03.021 16730429

[B62] Vaughn C. E. Leff J. (1976). The measurement of expressed emotion in the families of psychiatric patients. *Br. J. Soc. Clin. Psychol.* 15 157–165. 10.1111/j.2044-8260.1976.tb00021.x 938822

[B63] Vilardaga R. Hayes S. C. Atkins D. C. Bresee C. Kambiz A. (2013). Comparing experiential acceptance and cognitive reappraisal as predictors of functional outcome in individuals with serious mental illness. *Behav. Res. Ther.* 51 425–433. 10.1016/j.brat.2013.04.003 23747581 PMC3696417

[B64] Wells A. (2000). *Emotional Disorders and Metacognition: Innovative Cognitive Therapy.* Chichester: Wiley.

[B65] White R. Gumley A. McTaggart J. Rattrie L. McConville D. Cleare S. (2011). A feasibility study of Acceptance and Commitment Therapy for emotional dysfunction following psychosis. *Behav. Res. Ther.* 49 901–907. 10.1016/j.brat.2011.09.003 21975193

[B66] Wiedemann G. Rayki O. Feinstein E. Hahlweg K. (2002). The family questionnaire: development and validation of a new self-report scale for assessing expressed emotion. *Psychiatry Res.* 109 265–279. 10.1016/S0165-1781(02)00023-9 11959363

[B67] Wolthaus J. E. D. Dingemans P. M. A. J. Schene A. H. Linszen D. H. Wiersma D. Van Den Bosch R. J. (2002). Caregiver burden in recent-onset schizophrenia and spectrum disorders: the influence of symptoms and personality traits. *J. Nerv. Ment. Dis.* 190 241–247. 10.1097/01.NMD.0000012872.08131.50 11960085

